# The PRALINE database: protein and Rna humAn singLe nucleotIde variaNts in condEnsates

**DOI:** 10.1093/bioinformatics/btac847

**Published:** 2023-01-02

**Authors:** Andrea Vandelli, Magdalena Arnal Segura, Michele Monti, Jonathan Fiorentino, Laura Broglia, Alessio Colantoni, Natalia Sanchez de Groot, Marc Torrent Burgas, Alexandros Armaos, Gian Gaetano Tartaglia

**Affiliations:** Department of Biochemistry and Molecular Biology, Universitat Autònoma de Barcelona, Barcelona 08193, Spain; Universitat Pompeu Fabra (UPF), Barcelona 08003, Spain; Center for Human Technologies (CHT), Istituto Italiano di Tecnologia (IIT), Genova 16152, Italy; Department of Biology and Biotechnologies, University Sapienza Rome, Roma 00185, Italy; Center for Human Technologies (CHT), Istituto Italiano di Tecnologia (IIT), Genova 16152, Italy; Center for Human Technologies (CHT), Istituto Italiano di Tecnologia (IIT), Genova 16152, Italy; Center for Human Technologies (CHT), Istituto Italiano di Tecnologia (IIT), Genova 16152, Italy; Department of Biology and Biotechnologies, University Sapienza Rome, Roma 00185, Italy; Department of Biochemistry and Molecular Biology, Universitat Autònoma de Barcelona, Barcelona 08193, Spain; Department of Biochemistry and Molecular Biology, Universitat Autònoma de Barcelona, Barcelona 08193, Spain; Center for Human Technologies (CHT), Istituto Italiano di Tecnologia (IIT), Genova 16152, Italy; Center for Human Technologies (CHT), Istituto Italiano di Tecnologia (IIT), Genova 16152, Italy; Department of Biology and Biotechnologies, University Sapienza Rome, Roma 00185, Italy

## Abstract

**Summary:**

Biological condensates are membraneless organelles with different material properties. Proteins and RNAs are the main components, but most of their interactions are still unknown. Here, we introduce PRALINE, a database for the interrogation of proteins and RNAs contained in stress granules, processing bodies and other assemblies including droplets and amyloids. PRALINE provides information about the predicted and experimentally validated protein–protein, protein–RNA and RNA–RNA interactions. For proteins, it reports the liquid–liquid phase separation and liquid–solid phase separation propensities. For RNAs, it provides information on predicted secondary structure content. PRALINE shows detailed information on human single-nucleotide variants, their clinical significance and presence in protein and RNA binding sites, and how they can affect condensates’ physical properties.

**Availability and implementation:**

PRALINE is freely accessible on the web at http://praline.tartaglialab.com.

## 1 Introduction

Although the exact composition and functions of the different condensates are unknown, they are enriched in protein and RNA molecules that interact through protein–protein, protein–RNA and RNA–RNA networks. Solid-like condensates, and in particular amyloids, are generally considered to be inherently irreversible aberrant clumps (Dobson, 2017), while liquid-like condensates are dynamic entities that exchange components with the surrounding environment and grow, collapse and fuse in the nucleus and cytoplasm (Marchese *et al.*, 2016). Liquid-like condensates perform different functions on RNA molecules, such as storage in the germline, localization in neurons and protection from harmful conditions. The most known liquid-like condensates are processing bodies (PBs) and stress granules (SGs), both enriched in RNAs, which allow them to form and dissolve rapidly (Lorenzo Gotor *et al.*, 2020). Yet, subtle changes in the composition or concentration of condensates’ constituents can induce the formation of solid-like assemblies (Cid-Samper *et al.*, 2018). This is the case of Amyotrophic Lateral Sclerosis (ALS), where single-nucleotide variants (SNVs) in FUS trigger a liquid-to-solid phase transition (LSPT) (Patel *et al.*, 2015). Structural properties of the RNA and changes upon mutations are important, since they play a role in the process of condensation. Highly structured RNAs attract large amounts of proteins thanks to their intrinsic ability to establish stable interactions (Sanchez de Groot *et al.*, 2019). Moreover, RNAs can act as scaffolding elements (Armaos *et al.*, 2021), whereas a polypeptide of 100 amino acids can interact with one or two proteins, a chain of 100 nucleotides is able to bind to 5–20 proteins (Vandelli *et al.*, 2022). Poorly structured transcripts also induce condensation, as they base-pair with other RNAs establishing a dense network of contacts (Treeck *et al.*, 2018). All these data are gathered in PRALINE, a database that provides information on different condensates’ components, their interaction networks and disease-related variants.

## 2 Organization and content of the database

**Fig. 1.**
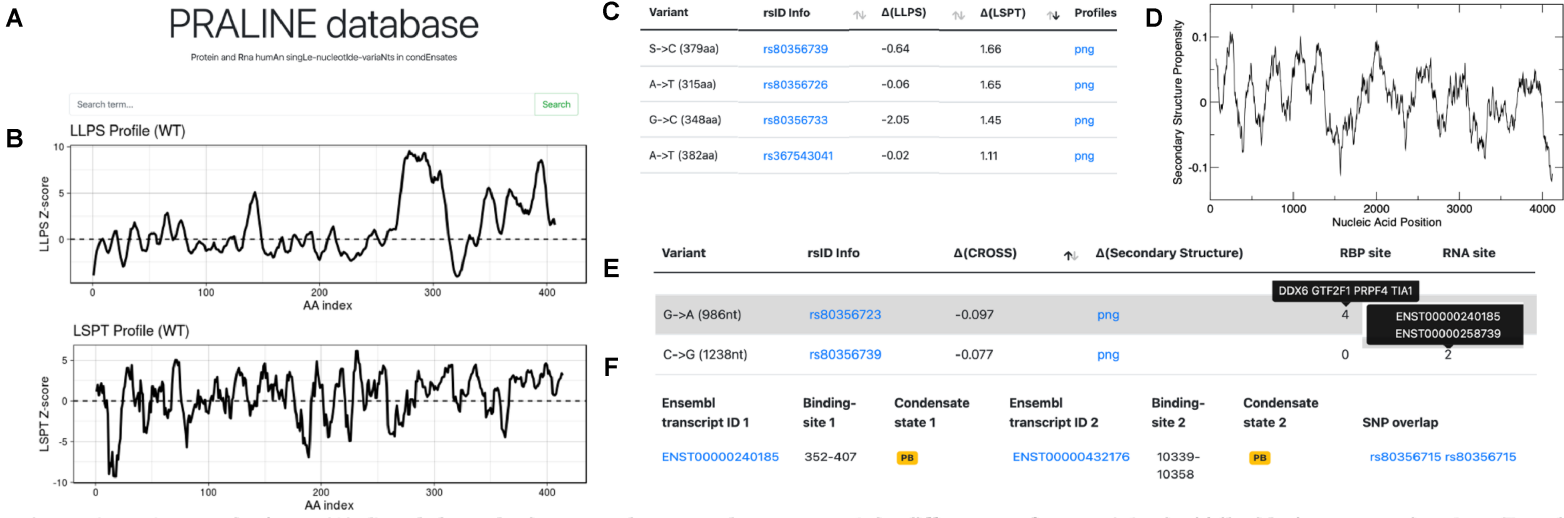
PRALINE database. (**A**) Search bar. The input can be a protein or an RNA in different ID formats. (**B**) Liquid–liquid phase separation (LLPS) and liquid–solid phase transition (LSPT) propensity profiles of a protein are predicted using *cat*GRANULE and Zyggregator algorithms. (**C**) Protein SNVs description table: the difference in LLPS and LSPT compared to the WT is provided. (**D**) CROSS secondary structure propensity profile image of a RNA sequence. (**E**) RNA SNVs description table: the difference in CROSS secondary structure propensity compared to the WT, corresponding to a 11-nt window around the mutation, is provided, as well as proteins and RNAs interacting with the query transcript containing the SNV. (**F**) Example of an RNA–RNA interaction table. The information about RNAs’ binding sites, condensates localization and SNVs falling inside at least one of the binding sites are reported. The examples **B–F** relate to *TARDBP*

PRALINE can be accessed using protein or RNA names provided as Gene Name, Ensembl Gene/Transcript ID (https://www.ensembl.org/) and UniprotKB ID (https://www.uniprot.org/; Fig. 1A).

- Searching for a specific protein, the user can retrieve information on the condensate state (droplet/liquid-like or amyloid/solid-like) and the organelle in which it has been found (SG/PB). The predicted liquid–liquid phase separation (LLPS) and LSPT propensities and profiles of the wild-type sequence are provided, calculated with *cat*GRANULE (Bolognesi *et al.*, 2016) and Zyggregator (Tartaglia *et al.*, 2008) methods, respectively (>0.80 accuracy in predicting regions of the proteins involved in protein condensation; Fig. 1B). Experimentally validated protein–protein interactions are available through links to BioGRID (https://thebiogrid.org/), while experimental and predicted protein–RNA interactions can be retrieved from RNAct (https://rnact.crg.eu/). Protein–RNA interactions are calculated using *cat*RAPID, an algorithm trained on NMR and X-ray structures (area under the curve (AUC), of 0.77 on eCLIP interactions) (Lang *et al.*, 2019). The number of SNVs is shown for the protein of interest and, for each SNV, it is possible to interrogate the amino acid position, the difference in LSPT and LLPS propensities compared to the reference (i.e. wild-type protein) and to retrieve information related to disease (Landrum *et al.*, 2014; Piñero *et al.*, 2020). LSPT and LLPS scores and profiles are provided (Fig. 1C).
- Searching for a specific RNA, the user can retrieve information on the condensate state (SG/PB), the RNA secondary structure content (table and profile predicted using CROSS, http://s.tartaglialab.com/page/cross_group), the experimentally validated RNA interactions (RISE database, http://rise.life.tsinghua.edu.cn/) and the predicted or experimentally validated protein interactions reported in RNAct (https://rnact.crg.eu/) for both the reference sequence and SNVs (Landrum *et al.*, 2014; Piñero *et al.*, 2020) (Fig. 1D and E). The RNA–RNA interactions table reports information on different binding partners, if the interactors belong to a condensate, binding sites location in the transcripts and related SNVs (Fig. 1F). The SNV section reports the position in the transcript, the difference in secondary structure compared to the reference (a numerical value and a profile image are provided) (Delli Ponti *et al.*, 2017), associated diseases and interactions with RNAs (RISE database) as well as proteins (eCLIP https://www.encodeproject.org/eclip/) that involve the SNV containing region (Fig. 1F).

For most genes, information is available at both the protein and RNA levels, so it is possible to navigate from one molecule to the other, revealing the links between them.

## 3 Applications

*PRALINE* is a database that provides a comprehensive view of protein and RNA interactions and SNVs in human liquid-like and solid-like condensates. Information about experimentally validated and predicted molecular interactions, including protein–protein, protein–RNA and RNA–RNA, is provided, as well as the predicted RNA secondary structure content and both LLPS and LSPT propensities of proteins.

For each SNV, we provide a description of the associated diseases, the binding sites and the change in RNA secondary structure, LLPS and LSPT propensities. Combining physico-chemical properties of molecules and disease-related annotations, PRALINE helps to unravel macromolecular connections that sustain different types of condensates and how variants can affect their equilibrium. PRALINE is the first database providing LLPS and LSPT predictions for SNVs, and we envisage that it would greatly facilitate the design of experiments to study condensates’ formation and implication in human diseases. Although tested extensively and validated experimentally, *cat*GRANULE predictions could not be benchmarked against a database of individual SNVs causing LLPS, due to a lack of adequate published resources. The availability of such databases will lead to a more precise understanding of the relationship between SNVs, structural conformations, protein–RNA assembly and diseases.

## Data Availability

General information is at https://praline.tartaglialab.com/about, where we provide a detailed description of the datasets and the tools employed in the database. Data provided in PRALINE are available at https://praline.tartaglialab.com/downloads. The tutorial is at https://praline.tartaglialab.com/tutorial.
